# Socioeconomic Factors Associated with Receipt of Minimally Invasive Surgery for NSCLC: Evidence from the National Cancer Database

**DOI:** 10.3390/cancers18040601

**Published:** 2026-02-12

**Authors:** Shama D. Karanth, Nimish Valvi, Mihika M. Shinde, Francesca Kowalik, Adaeze Aroh, Hiren J. Mehta, Michael K. Gould, Dejana Braithwaite

**Affiliations:** 1 Division of Population Health Sciences, Department of Surgery, University of Florida College of Medicine, Gainesville, FL 32610, USA; 2 University of Florida Health Cancer Center, University of Florida, Gainesville, FL 32608, USA; 3 College of Health, Department of Nutrition & Health Science, Ball State University, Muncie, IN 47306, USA; 4 College of Health Professions, Public Health Sciences, Slippery Rock University, Slippery Rock, PA 16057, USA; adaeze.aroh@sru.edu; 5 Division of Pulmonary, Critical Care and Sleep Medicine, University of Florida, Gainesville, FL 32610, USA; 6 Department of Health Systems Science, Kaiser Permanente Bernard J Tyson School of Medicine, Pasadena, CA 91101, USA

**Keywords:** lung cancer, minimally invasive resection, surgery, NSCLC

## Abstract

Background: Lung cancer remains the leading cause of cancer-related morbidity and mortality. Despite advances in surgical management, economically disadvantaged patients experience inequalities in the receipt of treatments. We evaluated the association between socioeconomic status (SES) and type of surgery: robotic-assisted thoracic surgery (RATS), video-assisted thoracic surgery (VATS), and open thoracotomy. Methods: Data came from the National Cancer Database (2015–2022) and included Stage 0–IIIa NSCLC patients. SES was measured by quartiles of median household income in the patient’s zip code. Adjusted multinomial logistic regression was used to estimate adjusted odds ratios (aORs) and 95% confidence intervals (CIs). Results: Among 84,931 patients with a mean age of 67.8 years, 38.4% underwent open thoracotomy, 33.3% underwent VATS, and 28.2% underwent RATS. Patients residing in the low-income areas (<$46,277) were significantly less likely to undergo RATS (aOR: 0.79, 95% CI: 0.77–0.86) or VATS (aOR: 0.62, 95% CI: 0.59–0.66) compared to patients living in high-income areas (≥$74,063). Community hospitals were less likely to provide RATS (aOR: 0.32, 95% CI: 0.29–0.35) or VATS (aOR: 0.58, 95% CI: 0.54–0.63) than academic centers. Conclusion: Socioeconomic disadvantage is associated with lower use of minimally invasive surgical approaches for NSCLC. Efforts to expand access to advanced surgical care may be necessary to reduce treatment disparities and improve outcomes.

## Simple Summary

It is not well understood how the neighborhood-level socioeconomic context shapes the likelihood of receiving minimally invasive surgical approaches, including RATS and VATS, for lung cancer. Using the National Cancer Database from 2015 to 2022, we analyzed 84,931 patients with non-small lung cancer (NSCLC) who underwent surgery. We compared three types of surgery: open thoracotomy, VATS, and RATS. Patients from the lowest-income neighborhoods were less likely to receive RATS or VATS compared to those from the highest-income areas, after adjusting for patient, clinical, and hospital factors. We also found that community hospitals were far less likely than academic centers to offer these advanced surgical techniques. Overall, the results show apparent socioeconomic differences in receipt of minimally invasive lung cancer surgery. Access to modern surgical care for patients in disadvantaged communities needs to be improved to reduce these treatment gaps and improve outcomes.

## 1. Introduction

Lung cancer remains the leading cause of cancer-related morbidity and mortality in the United States [1]. Among patients with early-stage non-small cell lung cancer (NSCLC), surgical resection offers the best chance for long-term survival [1,2]. Over the past two decades, minimally invasive approaches such as video-assisted thoracoscopic surgery (VATS) and robotic-assisted thoracoscopic surgery (RATS) have transformed the surgical management of early-stage NSCLC [3,4,5]. Both RATS and VATS procedures have demonstrated significant perioperative benefits over traditional open thoracotomy [5,6], including shorter length of stay, fewer complications, and potentially improved survival [7]. RATS, first introduced in the early 2000s, has gained extensive adoption in recent years, specifically since 2015 [8]. The rapid growth of RATS is partially driven by its technological advantages over VATS, including enhanced three-dimensional visualization, improved instrument dexterity, and better ergonomics [9]. Emerging evidence also suggests that RATS may be associated with lower intraoperative blood loss, higher lymph node retrieval rates, and reduced need for conversion to open surgery, making it an increasingly preferred option for anatomic lung resections [10,11].

Prior to the rise of RATS, the VATS procedure was established as a minimally invasive alternative to open thoracotomy and remains widely used in thoracic surgery [4,12]. Compared to open thoracotomy, VATS offers advantages, including reduced postoperative pain, lower complication rates, shorter hospital stays, and faster return to normal activity [13,14]. The broader availability of VATS-trained surgeons, lower equipment and maintenance costs, and greater accessibility across institutions contribute to its continued predominance over RATS in both academic and community settings [12,15].

Although adoption of RATS and VATS has expanded rapidly, open thoracotomy continues to be utilized, particularly for centrally located tumors, complex resections, re-operative cases, or in institutions with limited minimally invasive expertise or resources [16]. Current evidence demonstrates that older age, race, comorbidity burden, and socioeconomic factors, including both individual and neighborhood-level characteristics, significantly influence the receipt of minimally invasive surgery in early-stage NSCLC [17,18]. Consequently, the availability and utilization of these advanced surgical techniques may not be equitably distributed, raising important questions about access, system-level constraints, and broader social determinants that shape cancer care [19,20].

The purpose of this study was to evaluate the association between area-level socioeconomic factors, specifically median household income, and receipt of RATS, VATS, or open thoracotomy among patients with surgically managed NSCLC (Stages 0–IIIA) using contemporary data from the National Cancer Database (NCDB). We also assessed whether facility type and rurality modified these associations.

## 2. Materials and Methods

### 2.1. Data Source and Study Population

This study utilized data from the 2022 National Cancer Database Participant Use File (PUF). The NCDB is a nationwide, hospital-based clinical registry jointly sponsored by the American College of Surgeons’ Commission on Cancer (CoC) and the American Cancer Society (ACS) [21] (https://www.facs.org/quality-programs/cancer-programs/national-cancer-database/, accessed on 27 November 2025). It captures approximately 70% of all newly diagnosed cancer cases in the United States, making it one of the most comprehensive oncology datasets available, with over 34 million patient records to date. This analysis was conducted under the University of Florida’s Automated Determination Tools for Nonhuman and Exempt Research and did not require additional IRB approval. As this study involved secondary analysis of de-identified data, informed consent was not required.

**Figure 1:**
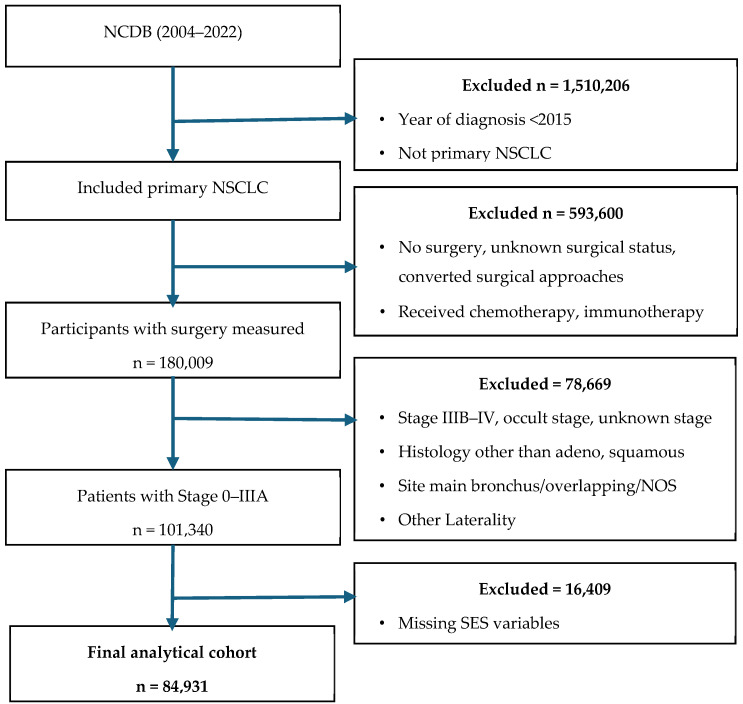
Flowchart of the study participants’ selection. Abbreviations: NCDB—National Cancer Database, NSCLC—non-small cell lung cancer, SES—socioeconomic status, NOS—not otherwise specified.

Data was drawn from the NCDB (2004–2022) to identify 2,283,815 patients diagnosed with NSCLC. Cases were identified using ICD-O-3 topography codes for lung and bronchus (C34.1–C34.3). NSCLC was defined using ICD-O-3 histology codes 8050, 8052, 8070–8075, 8083, 8140, 8141, 8144, 8250–8255, 8260, and 8310 [22]. We restricted to lobe-specific sites (C34.1–C34.3) to reduce heterogeneity in surgical management. To define the final cohort, we excluded patients diagnosed prior to 2015; those without lung as the primary tumor site; those who did not undergo surgery; those with Stage IIIB–IV, occult, or unknown stage disease; and those with a converted surgical approach. Converted procedures were identified using the NCDB’s predefined variable, which distinguishes robotic-assisted or minimally invasive procedures converted to open thoracotomy from primary open approaches. We restricted the cohort to patients diagnosed from 2015 onward to reflect contemporary surgical practice, including the rapid uptake of RATS in the United States [11,23]. Additional exclusions included cases with non-adenocarcinoma or non-squamous histology, tumors located in the bronchus or overlapping sites, and records with missing area-level socioeconomic status (SES) data. The resulting cohort comprised 84,931 patients with clinical stages 0–IIIA NSCLC who underwent anatomic resection via RATS, VATS, or open thoracotomy (Figure 1).

### 2.2. Surgical Procedures

The primary outcome of interest was surgical approach, categorized as RATS, VATS, or open thoracotomy. These categories were predefined according to NCDB procedure codes and reflect the operative technique used for definitive resection [21].

### 2.3. Area-Level Socioeconomic Factors

The primary exposure was area-level median household income. The income measures were derived by matching the patient’s zip code recorded at the time of diagnosis to files from the 2020 American Community Survey, spanning 2016–2020, and adjusted for 2020 inflation. Area-level median household income was categorized into quartiles (<$46,277, $46,227–$57,856, $57,857–$74,062, and ≥$74,063) [21]. Urban–rural residence was categorized as metropolitan, non-metropolitan, or missing. Facility characteristics included facility type (academic or community).

### 2.4. Covariates

Potential confounding factors were selected based on the literature and included demographic, geographic, clinical, and tumor-related characteristics. Demographics comprised age at diagnosis (<65, 65–74, ≥75 years), sex (male, female), race/ethnicity, and primary payer (private insurance, Medicare, Medicaid, uninsured, other government). Race/ethnicity was derived by combining the two NCDB variables “race” and “Spanish/Hispanic origin” and categorized as non-Hispanic (NH) White, NH-Black, Hispanic, NH-Asian, and NH-Other. Hereafter, the NH prefix is omitted when referring to racial/ethnic groups. The geographic region was categorized as Northeast, Midwest, South, or West. Clinical characteristics included the Charlson–Deyo comorbidity score (0, 1, 2, ≥3). Tumor characteristics included stage (0, IA, IB, IIA, IIB, IIIA), size (<2 cm, 2–3 cm, 3.1–4 cm, 4.1–5 cm, >5 cm), location (upper, middle, lower lobe), and laterality (right, left lung).

### 2.5. Statistical Analysis

Baseline characteristics of the study population were summarized and stratified by type of surgery. The variables were reported as counts (*n*) and percentages (%) and compared using Pearson’s chi-squared test for categorical variables. The association between area-level income and receipt of a specific type of surgery was evaluated using multivariable multinomial logistic regression, with the open approach as reference. Adjusted odds ratios (aORs) with 95% confidence intervals (CIs) and two-sided *p*-values < 0.05 were reported as statistically significant. Model fit was evaluated using deviance statistics and information criteria (AIC). Pearson and deviance chi-square goodness-of-fit tests were also examined. The final model demonstrated adequate fit (AIC = 181761.60; Pearson chi-square test, *p*-value = 0.0832). Predicted probabilities from the multinomial logistic regression model were calculated for each surgical approach and plotted across quartiles of median household income to visualize the association between socioeconomic status and receipt of RATS, VATS, or open thoracotomy. Predicted probabilities were derived from a multivariable multinomial logistic regression model and visualized using the SAS SGPLOT procedure (SAS version 9.4).

Subgroup analyses were conducted to evaluate the association between median household income and receipt of surgery type, stratified by facility type, race/ethnicity, and type of residence. Within each stratum, multivariable multinomial logistic regression was used to estimate adjusted odds ratios (aORs) (95% CI) for RATS versus open and VATS versus open, with open thoracotomy as the reference. All analyses were conducted using SAS 9.4 (SAS Institute, Cary, NC, United States).

## 3. Results

Among 84,931 patients with Stage 0–IIIA NSCLC in the NCDB (2015–2022) (Figure 1, Table 1), 23,932 (28.2%) underwent RATS, 28,358 (33.4%) VATS, and 32,641 (38.4%) open thoracotomies. The cohort had a mean (SD) age of 67.8 (8.5) years; 33.8% were aged < 65 years, 43.3% were 65–74 years, and 22.9% were ≥75 years. Females comprised 54.0% of the population. Most were White (81.6%), followed by Black (8.7%), Hispanic (3.6%), and Asian (3.4%); care was predominantly delivered in academic (96.1%) and metropolitan (79.2%) facilities. Medicare was the most common payer (62.9%), and 35.0% of the participants resided in the highest ZIP code income quartile (≥$74,063), compared with 17.6% in the lowest quartile (<$46,277). Regionally, 37.6% of the cases were treated in the South, 25.6% in the Midwest, 23.3% in the Northeast, and 13.5% in the West.

Clinically, 62.3% had Stage IA disease, and 68.5% had tumors ≤3 cm, most located in the upper lobe (61.2%). Compared to patients treated with open thoracotomy, those treated with RATS or VATS were more often treated at academic, metropolitan facilities and resided in higher-income and higher-education ZIP codes. Patients in the highest residential income quartile were more likely to undergo VATS (39.5%) or RATS (35.5%) than open thoracotomy (30.7%). Conversely, patients in the lowest-income quartile were more frequently treated with open thoracotomy (Table 1).

Residential median income was significantly associated with reduced receipt of minimally invasive surgery (RATS or VATS) relative to open thoracotomy (Table 2). Patients residing in the lowest-income quartile (<$46,277) were less likely to undergo RATS (aOR 0.79, 95% CI 0.77–0.86) or VATS (aOR 0.62, 95% CI 0.59–0.66) compared with those in the highest quartile (≥$74,063). Similarly, patients in the second quartile ($46,277–$57,856) had lower odds of receiving RATS (aOR 0.82, 95% CI 0.79–0.87) or VATS (aOR 0.69, 95% CI 0.66–0.72). There was no significant difference for RATS versus open thoracotomy among patients in the third quartile ($57,857–$74,062) (aOR 0.96, 95% CI 0.92–1.01), whereas VATS remained 22% less likely in this group (aOR 0.78, 95% CI 0.75–0.82). Patients treated at community facilities were significantly less likely to receive RATS (aOR 0.32, 95% CI 0.29–0.35) or VATS (aOR 0.58, 95% CI 0.54–0.63) compared to those in academic facilities.

Younger age (<65 years) was associated with a higher likelihood of receiving minimally invasive surgery (*p* < 0.001), and males had slightly lower odds compared to females (*p* = 0.0002). Racial and ethnic differences were observed, with Black, Hispanic, and Asian patients more likely to receive RATS compared with White patients, though these patterns were not consistent for VATS. An increasing Charlson–Deyo score ≥ 1 was associated with higher odds of VATS and RATS compared with open thoracotomy (*p* < 0.001). Insurance status also influenced the surgical approach. Uninsured patients were significantly less likely to undergo RATS (aOR 0.81, 95% CI 0.68–0.95), while those with other government insurance were less likely to receive VATS (aOR 0.81, 95% CI 0.69–0.95) compared with privately insured patients (Table 2).

**Figure 2:**
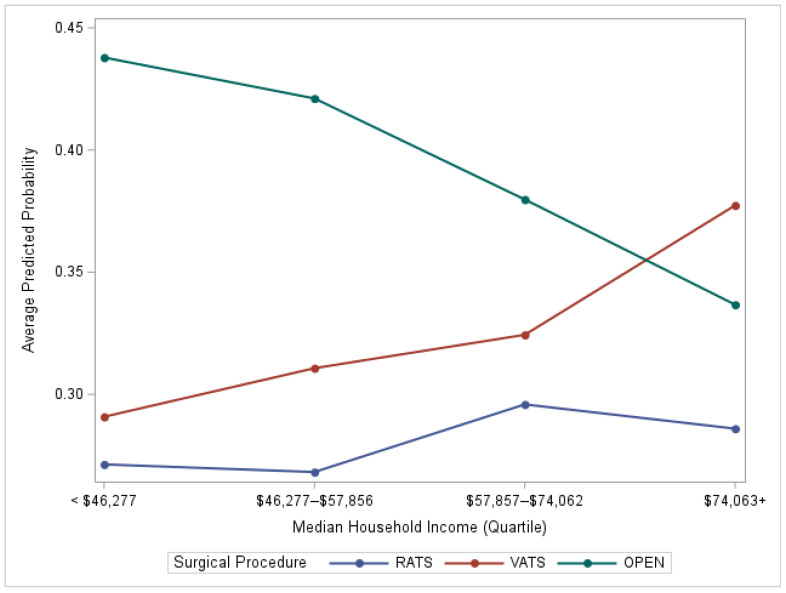
Model-estimated probability of RATS and VATS among NSCLC patients across income levels. The y-axis represents the model-estimated probability of receipt of each surgery type, illustrating how predicted likelihood varies by median household income quartiles. Abbreviation: RATS—robotic-assisted thoracoscopic surgery; VATS—video-assisted thoracoscopic surgery.

Model-estimated probabilities of receiving RATS, VATS, or open thoracotomy varied across income quartiles (Figure 2). The probability of undergoing open thoracotomy declined with increasing income, while the likelihood of VATS and, to a lesser extent, RATS increased among patients residing in higher-income areas.

**Figure 3:**
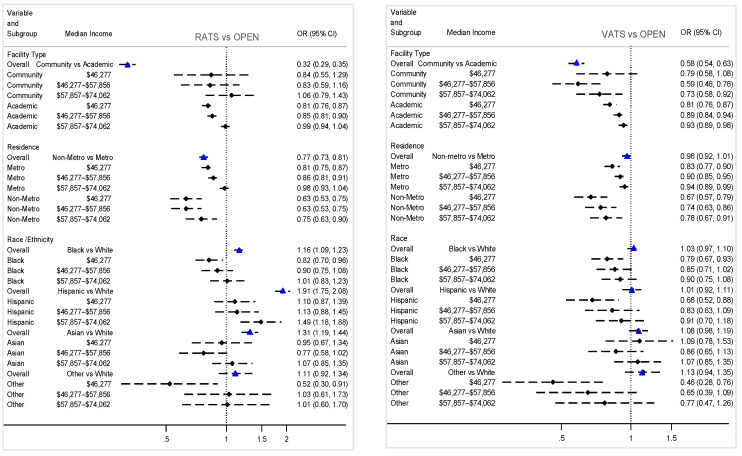
Subgroup analysis of the association between surgical approach and median household income, National Cancer Database, 2015–2022. Abbreviations: RATS—robotic-assisted thoracoscopic surgery; VATS—video-assisted thoracoscopic surgery. Reference for income ≥$74,063. Interaction *p*-values for facility type × income (*p* = 0.0004), residence × income (*p* ≤ 0.0001), and race × income (*p* = 0.0337). 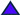
—indicates the overall odds ratio for each variable category.

In subgroup analyses (Figure 3), residing in a lower-income ZIP code (compared with ≥$74,063) was consistently associated with lower use of minimally invasive surgery, although the pattern differed by care setting (*p*-interaction <0.001). In community facilities, receipt of VATS declined across all lower-income quartiles, while RATS showed no significant income-related differences. In academic centers, patients from lower-income areas were less likely to receive RATS in the two lowest quartiles and less likely to receive VATS across all three lower-income quartiles. Similarly, effect modification by residence (*p*-interaction < 0.0001) revealed that in metropolitan areas, lower-income patients were less likely to receive RATS or VATS compared with higher-income patients, with the steepest decline observed for VATS. In non-metro areas, the socioeconomic gradient was even more pronounced, with substantially fewer patients in all lower-income quartiles receiving minimally invasive surgery. There was a significant interaction between median household income and race/ethnicity (*p*-interaction = 0.03). Although overall, Black, Hispanic, and Asian patients were more likely to receive RATS compared with White patients, these associations were attenuated in subgroup analyses. Patients from lower-income areas were less likely to receive both RATS and VATS, indicating that the surgical approach varied across income strata (Figure 3).

## 4. Discussion

### 4.1. Overview of Findings

In this national cohort of 84,931 surgically treated primary NSCLC patients, minimally invasive approaches were frequently recorded but unevenly distributed, particularly among patients residing in lower-income areas. Patients who received RATS or VATS were more often treated at academic, metropolitan centers and resided in higher-income ZIP codes. They also tended to have earlier-stage and smaller tumors, reflecting both the concentration of advanced surgical technology and the selection of anatomically favorable cases. Our findings underscore the impact of income, insurance status, and residence on disparities in surgical access.

Prior studies have reported socioeconomic disparities in the adoption of minimally invasive lung surgeries; most have combined RATS and VATS or relied on older datasets [8,17,24]. Building on this literature, our study updates the evidence by evaluating RATS and VATS as distinct surgical modalities in a contemporary national cohort, revealing differential sociodemographic and clinical patterns in their use. Our findings reinforce previously observed associations [8,24] between area-level income and access to oncology treatments, particularly receipt of RATS.

Socioeconomic factors in access to robotic surgery have also been reported to influence other cancer types [24,25,26,27]. Patients residing in more deprived neighborhoods may face barriers such as distance or experience lower access to hospitals with the necessary resources to provide costly robotic surgery. Our findings support the established trends that patients from lower-income areas are less likely to receive robotic surgery and more likely to receive open thoracotomy for lung cancer.

In addition to area-based income, we also found strong associations for race/ethnicity, insurance status, facility type, and metropolitan residence. Patients living in non-metropolitan areas face geographic isolation and reduced access to primary and limited specialty care [28,29], and hospitals with fewer resources were far less likely to receive RATS or VATS compared with open thoracotomy [30]. These structural factors align with our findings that non-metropolitan patients were far less likely to receive minimally invasive procedures (RATS or VATS) rather than open thoracotomy, supporting other studies on treatment disparities by residence [28,29,30,31]. Patients residing in lower-income neighborhoods in our cohort were less likely to undergo RATS or VATS, likely due to limited access to hospitals with advanced resources and trained thoracic surgeons. While overall use of RATS and VATS was higher among Black, Hispanic, and Asian patients compared with White patients [18], stratified analyses by race/ethnicity and neighborhood income revealed that racial and ethnic minority patients in lower-income areas remained less likely to receive minimally invasive procedures, highlighting persistent inequities in surgical care.

Our results align with a prior analysis that examined combined RATS and VATS versus open thoracotomy using NCDB data from 2010 to 2018 [8], which found that patients from lower-income neighborhoods were significantly less likely to receive minimally invasive surgery. By evaluating RATS and VATS separately, our study provides further insight, revealing that robotic- and video-assisted procedures are particularly limited in lower-income areas, community facilities, and non-metropolitan centers. Consequently, the lower receipt of RATS/VATS compared with open thoracotomy resections among patients from lower-income neighborhoods likely reflects differences in both institutional resources and the distribution of specialized surgical expertise [20]. Academic centers often employ thoracic surgeons, who are more likely than general surgeons to perform minimally invasive lobectomies, which may explain differences in surgical approach across hospital types [20].

### 4.2. Clinical and Policy Implications

Our findings highlight persistent disparities in access to minimally invasive lung cancer surgery, particularly robotic and video-assisted approaches, among patients from lower-income neighborhoods, community hospitals, and non-metropolitan centers. Because RATS and VATS are associated with lower perioperative morbidity, lower infections, shorter recovery, and comparable oncologic outcomes to open thoracotomy, these disparities may contribute to avoidable differences in patient outcomes [6,14,32]. Clinicians should consider these inequities when counseling patients and, when appropriate, refer eligible individuals to high-volume or specialized centers to optimize surgical care. From a policy perspective, expanding access to minimally invasive surgical technology, increasing training opportunities for thoracic surgeons, and ensuring equitable insurance coverage could help reduce these gaps [19,20]. Structured referral networks and targeted resource allocation may further promote equitable adoption of advanced surgical approaches [33].

### 4.3. Strengths and Limitations

Strengths of this study include its large, nationally representative cohort and detailed characterization of surgical modality. Our study also has several limitations. We used area-level income measures, which may not accurately reflect individual-level income. However, area-based indicators capture broader contextual influences on access to technology and healthcare delivery, which often shape the receipt of treatment. Even after adjusting for clinical and sociodemographic variables, unmeasured factors such as patient preference, surgeon experience, or hospital-specific protocols may still influence the surgical approach. The NCDB does not collect data on certain clinical indices, like forced expiratory volume, performance status, smoking history, body mass index, or detailed comorbidity information beyond a pre-calculated comorbidity index [21]. Consequently, the study does not capture critical patient-level determinants, including individual misconceptions concerning or refusal of minimally invasive procedures, which may contribute to residual confounding and treatment selection.

## 5. Conclusions

In this retrospective national study, we observed that patients from lower-income areas, those who were uninsured, and those treated at community facilities were less likely to receive RATS and VATS procedures. Minimally invasive surgical approaches are associated with lower perioperative morbidity, faster recovery, and comparable oncologic outcomes, and our findings indicate that disparities in access to these approaches persist. These results suggest that technological advances alone may not be sufficient to reduce differences and highlight the importance of continued efforts to improve equitable access to minimally invasive and robotic surgical care. Policy efforts to reduce inequities in access should focus on improving the availability of high-quality surgical care and ensuring that all patients, regardless of socioeconomic status or geographic location, can benefit from minimally invasive approaches. Future research should investigate the long-term effects of surgical disparities on patient outcomes and test interventions, policy changes, and patient-centered strategies to ensure equitable access to minimally invasive thoracic surgery.

## Figures and Tables

**Table 1:** Sociodemographic and cancer characteristics stratified by surgery type, National Cancer Database, 2015–2022.

Variables	Overall	Robotic-Assisted (RATS) Surgery	Video-Assisted (VATS) Surgery	Open Surgery
	*n* = 84,931	*n* = 23,932	*n* = 28,358	*n* = 32,641
Age, mean (SD) *	67.8 (8.5)	68.2 (8.4)	68.1 (8.6)	67.3 (8.6)
<65	28,714 (33.8)	7686 (32.1)	9237 (32.6)	11,791 (36.1)
65–74	36,736 (43.3)	10,495 (43.7)	12,403 (43.7)	13,838 (42.4)
≥75	19,481 (22.9)	5751 (24.0)	6718 (23.7)	7012 (21.5)
**Female**	45,864 (54.0)	13,220 (55.2)	15,680 (55.3)	16,964 (52.0)
**Race**				
White	69,330 (81.6)	18,905 (79.0)	23,482 (82.8)	26,943 (82.5)
Black	7363 (8.7)	2207 (9.2)	2280 (8.0)	2876 (8.8)
Hispanic	3053 (3.6)	1289 (5.4)	805 (2.8)	959 (2.9)
Asian	2849 (3.4)	936 (3.9)	964 (3.4)	949 (2.9)
Other	680 (0.8)	189 (0.8)	238 (0.8)	253 (0.8)
Missing	1656 (2.0)	406 (1.7)	589 (2.1)	661 (2.0)
**Charlson–Deyo Score**				
None	45,263 (53.3)	12,795 (53.5)	14,874 (52.5)	17,594 (53.9)
1 condition	23,416 (27.6)	6644 (27.8)	7834 (27.6)	8938 (27.4)
2 conditions	9585 (11.3)	2570 (10.7)	3336 (11.8)	3679 (11.3)
≥3 conditions	6667(7.9)	1923 (8.0)	2314 (8.2)	2430 (7.4)
**Primary Payer**				
Not insured	928 (1.1)	221 (0.9)	263 (0.9)	444 (1.4)
Private insurance/managed care	22,952 (27.0)	6232 (26.0)	7696 (27.1)	9024 (27.7)
Medicaid	5319 (6.3)	1378 (5.8)	1731 (6.1)	2210 (6.8)
Medicare	53,420 (62.9)	15,457 (64.6)	18,017 (63.5)	19,946 (61.1)
Other government	1553 (1.8)	462 (1.9)	419 (1.5)	672 (2.1)
Missing	759 (0.9)	182 (0.8)	232 (0.8)	345 (1.1)
**Facility type**				
Community	3351 (4.0)	465 (1.9)	991 (3.5)	1895 (5.8)
Academic	81,580 (96.1)	23,467 (98.1)	27,367 (96.5)	30,746 (94.2)
**Median Income Quartiles**				
<$46,277	14,923 (17.6)	4049 (16.9)	4340 (15.3)	6534 (20.0)
$46,227–$57,856	19,366 (22.8)	5194 (21.7)	6017 (21.2)	8155 (25.0)
$57,857–$74,062	20,924 (24.6)	6191 (25.9)	6787 (23.9)	7946 (24.3)
≥$74,063	29,718 (35.0)	8498 (35.5)	11,214 (39.5)	10,006 (30.7)
**Urban/Rural**				
Metro	67,224 (79.2)	19,583 (81.8)	22,585 (79.6)	25,056 (76.8)
Non-Metro	14,649 (17.3)	3405 (14.2)	4743 (16.7)	6501 (19.9)
Missing	3058 (3.6)	944 (3.9)	1030 (3.6)	1084 (3.3)
**Location of facility**				
Northeast	19,756 (23.3)	5252 (22.0)	8705 (30.7)	5799 (17.8)
Midwest	21,752 (25.6)	6048 (25.3)	5572 (19.7)	10,132 (31.0)
West	11,499 (13.5)	2769 (11.6)	4635 (16.3)	4095 (12.6)
South	31,924 (37.6)	9863 (41.2)	9446 (33.3)	12,615 (38.7)
**Primary Site**				
Upper lobe	51,971 (61.2)	14,286 (59.7)	17,283 (61.0)	20,402 (62.5)
Mid-lobe	4318 (5.1)	1288 (5.4)	1497 (5.3)	1533 (4.7)
Lower Lobe	28,642 (33.7)	8358 (34.9)	9578 (33.8)	10,706 (32.8)
**Laterality**				
Right	50,118 (59.0)	14,439 (60.3)	16,754 (59.1)	18,925 (58.0)
Left	34,813 (41.0)	9493 (39.7)	11,604 (40.9)	13,716 (42.0)
**Stage**				
0	760 (0.9)	198 (0.8)	300 (1.1)	262 (0.8)
IA	52,877 (62.3)	16,187 (67.6)	18,549 (65.4)	18,141 (55.6)
IB	12,114 (14.3)	3163 (13.2)	3928 (13.9)	5023 (15.4)
IIA	5598 (6.6)	1197 (5.0)	1658 (5.9)	2743 (8.4)
IIB	7009 (8.3)	1744 (7.3)	2003 (7.1)	3262 (10.0)
IIIA	6573 (7.7)	1443 (6.0)	1920 (6.8)	3210 (9.8)
**Tumor size**				
<2 cm	32,802 (38.6)	9825 (41.1)	12,126 (42.8)	10,851 (33.2)
2 to 3 cm	25,358 (29.9)	7553 (31.6)	8273 (29.2)	9532 (29.2)
3.1 to 4 cm	11,449 (13.5)	3207 (13.4)	3581 (12.6)	4661 (14.3)
4.1 to 5 cm	6215 (7.3)	1551 (6.5)	1867 (6.6)	2797 (8.6)
>5 cm	9107 (10.7)	1796 (7.5)	2511 (8.9)	4800 (14.7)

* Abbreviations: SD—standard deviation.

**Table 2:** Factors associated with receipt of type of surgery among patients with Stage 0–IIIA non-small cell lung cancer (NSCLC), National Cancer Database, 2015–2022 (*n* = 84,931).

Variable	* RATS vs. Open aOR (95% CI)	*P*-Value	* VATS vs. Open aOR (95% CI)	*P*-Value
**Median Household Income**		<0.001		<0.001
<$46,277 vs. ≥$74,063	0.79 (0.77, 0.86)		0.62 (0.59, 0.66)	
$46,277–$57,856 vs. ≥$74,063	0.82 (0.79, 0.87)		0.69 (0.66, 0.72)	
$57,857–$74,062 vs. ≥$74,063	0.96 (0.92, 1.01)		0.78 (0.75, 0.82)	
**Facility type**		<0.0001		<0.0001
Community vs. academic	0.32 (0.29, 0.35)		0.58 (0.54, 0.63)	
**Sex**		0.0002		0.0002
Male vs. female	0.94 (0.91, 0.98)		0.93 (0.90, 0.96)	
**Age**		<0.0001		<0.0001
<65 vs. ≥75	1.11 (1.06, 1.16)		1.11 (1.07, 1.16)	
65–74 vs. ≥75	1.21 (1.15, 1.28)		1.19 (1.13, 1.25)	
**Race/ethnicity**		<0.0001		<0.0001
Black vs. White	1.16 (1.09, 1.23)		1.03 (0.97, 1.10)	
Asian vs. White	1.31 (1.19, 1.44)		1.08 (0.98, 1.18)	
Hispanic vs. White	1.91 (1.75, 2.08)		1.01 (0.92, 1.11)	
Other vs. White	1.11 (0.92, 1.35)		1.13 (0.94, 1.35)	
Unknown vs. White	0.91 (0.80, 1.03)		1.35 (1.12, 1.65)	
**Charlson–Deyo Score**		<0.0001		<0.0001
1 vs. 0	1.03 (0.99, 1.08)		1.06 (1.02, 1.10)	
2 vs. 0	0.98 (0.93, 1.04)		1.10 (1.05, 1.16)	
3 ≥ vs. 0	1.08 (1.01, 1.16)		1.15 (1.08, 1.23)	
**Primary payer**		<0.0001		<0.0001
Not insured vs. private	0.81 (0.68, 0.95)		0.81 (0.69, 0.95)	
Government vs. private	1.04 (0.99, 1.09)		0.99 (0.94, 1.03)	
Unknown vs. private	0.73 (0.61, 0.88)		0.77 (0.65, 0.91)	
**Urban/rural**		<0.0001		<0.0001
Non-metro vs. metro	0.77 (0.73, 0.81)		0.96 (0.92, 1.01)	
**Primary site**		<0.0001		<0.0001
Mid-lobe vs. upper lobe	1.10 (1.02, 1.19)		1.09 (1.01, 1.18)	
Lower lobe vs. upper lobe	1.14 (1.10, 1.19)		1.08 (1.04, 1.11)	
**Laterality**		<0.0001		<0.0001
Left vs. right	0.92 (0.89, 0.95)		0.97 (0.94, 1.01)	
**Stage**		<0.0001		<0.0001
1 vs. 0	1.20 (0.99, 1.44)		0.94 (0.79, 1.11)	
2 vs. 0	0.93 (0.77, 1.13)		0.75 (0.63, 0.90)	
3 vs. 0	0.83 (0.68, 1.02)		0.72 (0.60, 0.86)	
**Tumor size**		<0.0001		<0.0001
2 to 3 cm vs. <2 cm	0.89 (0.86, 0.93)		0.80 (0.77, 0.83)	
3.1 to 4 cm vs. <2 cm	0.80 (0.77, 0.83)		0.72 (0.69, 0.76)	
4.1 to 5 cm vs. <2 cm	0.69 (0.64, 0.74)		0.67 (0.63, 0.72)	
>5 cm vs <2 cm	0.51 (0.47, 0.55)		0.57 (0.53, 0.61)	

* Abbreviation: RATS—robotic-assisted thoracoscopic surgery; VATS—video-assisted thoracoscopic surgery; NSCLC—non-small cell lung cancer.

## Data Availability

Data was de-identified and obtained from the NCDB via [ACS/CoC approval process].
